# Arthropod Exposure-Associated Neurogenic Pruritus: A Complex Neuro-Immune Transition Mimicking Refractory Urticaria

**DOI:** 10.7759/cureus.107918

**Published:** 2026-04-28

**Authors:** Niyas Khalid Ottu Para, Seema Rab

**Affiliations:** 1 Internal Medicine, Burjeel Hospital, Abu Dhabi, ARE; 2 Internal Medicine, Burjeel Holdings, Abu Dhabi, ARE

**Keywords:** allergic urticaria, arthropod exposure, chronic urticaria, immune urticaria, neurogenic pruritus

## Abstract

Chronic pruritus is a complex and often multifactorial clinical condition that may evolve beyond its initial inflammatory trigger, leading to significant diagnostic and therapeutic challenges. We report the case of a 45-year-old female who developed a persistent and progressively refractory pruritic disorder following arthropod exposure, clinically consistent with bedbug exposure. The condition evolved from an acute inflammatory eruption into a chronic state characterized by severe itch, evolving lesion morphology, and incomplete response to antihistamines and corticosteroids despite stepwise escalation, including leukotriene receptor antagonists and prolonged corticosteroid therapy. The presence of ecchymotic lesions early in the disease course prompted extensive hematologic and autoimmune evaluation, which was unremarkable. Over time, the clinical pattern shifted toward a neurogenic phenotype with strong psychophysiologic modulation, particularly in relation to stress. The patient demonstrated significant improvement following initiation of neuromodulatory therapy with gabapentin and mirtazapine. This case emphasizes the clinical importance of early recognition of a clinically inferred neuroimmune transition to avoid ineffective therapeutic escalation and enable mechanism-based, targeted neuromodulatory management in refractory cases.

## Introduction

Chronic pruritus is increasingly recognized as a multidimensional disorder involving complex interactions between the skin, immune system, nervous system, and psychological state. Contemporary reviews emphasize that persistent itch is not solely a cutaneous inflammatory phenomenon but can reflect integrated neuroimmune dysregulation, with major clinical implications for diagnosis and treatment. In many patients, the dominant biology shifts over time from peripheral inflammatory signalling to centrally amplified, non-histaminergic itch pathways, rendering conventional antihistamine-centered approaches inadequate [1,2].

The modern biology of chronic itch has highlighted the importance of cytokines and alarmins such as interleukin-31 (IL-31), thymic stromal lymphopoietin (TSLP), and type 2 inflammatory mediators, together with transient receptor potential channels, such as TRPV1 and TRPA1, in sustaining chronic pruritus independently of classical histamine pathways. These pathways form part of bidirectional neuroimmune circuits in which epidermal immune activation, peripheral sensory nerves, spinal processing, and higher-order emotional and attentional networks reinforce one another [1,3,4]. While these mechanisms are well-established in the literature, their role in this case is inferred from the clinical trajectory and treatment response.

Arthropod bite reactions are usually self-limited. But in some individuals, like other cutaneous inflammatory insults, they may act as triggering events for the dysregulation of itch pathways. The transition from an acute inflammatory response to chronic neurogenic pruritus remains under-recognized in practice, and such cases are often managed as refractory urticaria, with repeated escalation of antihistamines and corticosteroids despite limited and short-lived benefit [2,5].

Psychophysiologic factors can further amplify this process. Chronic stress, sleep disruption, neurocognitive vulnerability, and hormonal transition states, such as perimenopause, all have plausible effects on itch perception, skin barrier integrity, and neuroimmune signalling. Emerging literature also suggests overlap between chronic pruritus, attention-deficit/hyperactivity disorder (ADHD), and skin-picking behaviour, raising the possibility that certain patients may be biologically predisposed to sensory amplification and itch-scratch loop reinforcement, as supported by emerging and hypothesis-generating evidence [6,7].

We present a case that demonstrates this transition in a clinically striking way and highlights the importance of a mechanism-based, multidisciplinary approach to chronic pruritus.

## Case presentation

A 45-year-old female with a background of ADHD, a high-demand, executive professional role involving frequent travel, and significant ongoing psychosocial stress presented with a persistent and progressively debilitating pruritic disorder of approximately 4 months duration. The patient was menopausal and had been on hormone replacement therapy (HRT) prior to the onset of symptoms, which she discontinued during the disease course due to concern for a possible drug-related exacerbation. The illness began acutely following a hotel stay during which the patient sustained multiple arthropod bites, with an initial localized eruption consisting of discrete erythematous papules over exposed areas, clinically consistent with bedbug bite reactions. Over the subsequent days, the eruption progressed beyond the initial bite sites, becoming diffuse and generalized, involving the bilateral lower limbs and thighs. The distribution no longer followed a linear or clustered (eg, ‘breakfast-lunch-dinner’) pattern typically described with bedbug bites. As the disease evolved, the morphology transitioned from urticarial-like lesions to persistent pruritic areas with excoriations and post-inflammatory hyperpigmentation, reflecting a shift away from a localized inflammatory reaction toward a more widespread and chronic pruritic process.

Following discharge, the patient entered a fluctuating but progressively worsening course. She developed recurrent episodes of pruritus with evolving morphology and distribution. Initially, the lesions were urticaria-like, with intermittent wheal formation and diffuse erythema. However, over time, the clinical pattern became less consistent with classical urticaria. The lesions evolved into persistent pruritic areas, excoriated papules, and post-inflammatory skin changes. The itch progressively increased in both intensity and persistence, transitioning from episodic to near continuous.

The severity of pruritus became a defining feature of the illness. The patient described the itch as intrusive, overwhelming, and difficult to suppress, often leading to compulsive, urge-driven scratching. This resulted in multiple excoriations and, at times, bleeding lesions. She reported episodes of blood staining on her pillow due to nocturnal scratching, reflecting both severity and loss of behavioral control during sleep. Sleep disturbance became prominent and further compounded her overall symptom burden.

An additional atypical feature early in the disease course was the development of ecchymotic lesions in association with the rash. This raised concern for possible vasculitic, hematologic, or coagulation abnormalities and prompted a broadened diagnostic evaluation. Comprehensive investigations were undertaken, including coagulation profile, protein C, protein S, von Willebrand factor, and clotting factor assays, which were largely within normal limits except for a borderline reduction in factor VIII. Thrombophilia screening, including factor V Leiden mutation and antiphospholipid antibodies, was negative. Autoimmune evaluation, including antinuclear antibodies, anti-double-stranded DNA, antineutrophil cytoplasmic antibodies, and extractable nuclear antigen panel, was unremarkable. Inflammatory markers were within normal limits, and total IgE levels were not elevated. Overall, an extensive systemic workup did not reveal any clinically significant underlying autoimmune, vasculitic, allergic, or primary hematologic pathology to explain the patient’s presentation, despite minor non-specific laboratory deviations, including mild anemia and borderline coagulation parameter deviations. A comprehensive summary of laboratory and diagnostic investigations is presented in Table 1.

**Table 1:** Summary of laboratory and diagnostic investigations Abbreviations: MCV: Mean corpuscular volume; MCH: Mean corpuscular hemoglobin; RDW: Red cell distribution width; WBC: White blood cell count; CRP: C-reactive protein; ALT: Alanine aminotransferase; AST: Aspartate aminotransferase; ANA: Antinuclear antibody; ENA: Extractable nuclear antigen; ANCA: Antineutrophil cytoplasmic antibody; Anti-dsDNA: Anti-double-stranded DNA antibody; vWF: von Willebrand factor.

Test	Patient Value	Units	Reference Range
Hemoglobin	10.1–11.2	g/dL	12–16
Hematocrit	31.8–33.9	%	36–46
MCV	79–80	fL	80–100
MCH	25–26	pg	27–33
RDW	14.9	%	11.5–14.5
WBC	8.4–10.0	×10^9/L	4–11
Neutrophils	82–86%	%	40–70
Lymphocytes	10–24%	%	20–40
Platelets	203–245	×10^9/L	150–450
CRP	0.6–2.93	mg/L	<5
Sodium	135–136	mmol/L	135–145
Potassium	4.4–4.6	mmol/L	3.5–5.1
Creatinine	47–54	µmol/L	45–90
ALT	14–40	U/L	<35
AST	20–46	U/L	<35
Ferritin	32–38	µg/L	15–150
Protein C	148	%	70–140
Protein S	59	%	60–130
Factor VIII	56–71	%	60–150
vWF Activity	105	%	50–200
vWF Antigen	101	%	50–160
Factor V Leiden	Negative	-	Negative
ANA / ENA / ANCA	Negative	-	Negative
Anti-dsDNA	Negative	-	Negative
Anticardiolipin	Negative	-	Negative
Beta-2 Glycoprotein	Negative	-	Negative

Therapeutically, the patient underwent a prolonged and stepwise escalation of treatment, reflecting the refractory nature of her condition. Initial management consisted of high-dose second-generation antihistamines, including fexofenadine and levocetirizine, administered in combination and at escalated doses. Due to inadequate response, therapy was expanded to include an H2 receptor blockade and montelukast, targeting a broader anti-allergic and leukotriene-mediated pathway. Despite this extensive regimen, the patient continued to experience severe pruritus with only minimal and inconsistent relief.

Given the persistence and severity of symptoms, multiple courses of systemic corticosteroids were administered. While transient improvement was achieved during higher-dose therapy, symptoms consistently recurred during tapering. A prolonged tapering course over approximately six weeks was undertaken; however, this again resulted in only temporary suppression of symptoms without sustained remission. At this stage, the disease could be clearly characterized as refractory, having failed maximal antihistamine therapy, H2 blockade, leukotriene receptor antagonism, and prolonged corticosteroid treatment.

Dermatology consultation was obtained, and continuation of antihistamine-based therapy with corticosteroids was advised; however, this approach did not result in meaningful improvement. A skin biopsy was not performed, as the lesions were diffuse, evolving, and predominantly excoriation-modified, without features suggestive of a specific primary dermatosis such as vasculitis, blistering disease, or infiltrative pathology. It was therefore felt that a biopsy would likely yield nonspecific findings and would not alter management. Over time, the clinical phenotype evolved further, with diminishing urticarial features and increasing predominance of excoriated papules, prurigo-like lesions, and persistent background pruritus. The distribution of lesions increasingly corresponded to areas accessible to scratching, suggesting a well-established itch-scratch cycle.

During the course of treatment, the patient was initiated on gabapentin, with a rapid and notable initial improvement in pruritus within the first two to three days. However, this response was not sustained, and symptoms subsequently fluctuated, with partial recurrence of itch intensity. Around this period, the patient also developed a transient flare characterized by increased rash and pruritus, temporally associated with the use of gabapentin and concurrent cefuroxime prescribed for an intercurrent throat infection. Given the diagnostic uncertainty regarding a possible drug-related exacerbation, gabapentin was temporarily withheld; however, the pruritus persisted despite discontinuation, suggesting that the flare was unlikely to be solely medication-induced.

A critical and reproducible feature of the illness was the strong psychophysiologic modulation of symptoms. The patient consistently reported exacerbation of pruritus during periods of emotional distress, occupational stress, frequent travel, and interpersonal strain. She explicitly described that her rash and itch worsened when she was “not okay,” and this pattern was repeatedly observed over time. Her baseline context included a highly demanding professional environment, frequent travel, disrupted sleep patterns, and ongoing interpersonal stress, all of which appeared to correlate closely with disease activity.

Given the lack of sustained response to maximal anti-allergic therapy, including antihistamines, H2 blockade, montelukast, and corticosteroids, combined with the evolving morphology, partial initial response to gabapentin followed by fluctuation in symptom control and strong psychophysiologic correlation, the working diagnosis was reconsidered. The clinical picture was felt to be more consistent with a neurogenic and centrally amplified pruritic disorder rather than persistent histamine-mediated urticaria.

At this stage, escalation to biologic therapy, including agents such as omalizumab and dupilumab, was considered, given the refractory nature of symptoms. However, in view of the evolving phenotype, lack of sustained response to anti-allergic strategies, and increasing evidence of a centrally mediated mechanism, a decision was made to defer biologic therapy and instead pursue a neuro-modulatory approach.

Gabapentin 300 mg was reintroduced and subsequently titrated based on clinical response, and mirtazapine 30 mg was subsequently added, particularly targeting nocturnal symptoms, sleep disturbance, and associated psychological stress. Following this combination, the patient experienced more sustained and clinically significant improvement, starting within the first week and improving steadily over the next two to three weeks, with a reduction in both severity and frequency of pruritic episodes.

At the time of the most recent follow-up, the patient demonstrated significant overall improvement. Residual symptoms persisted and were closely linked to psychological stress. Notably, the patient reported a rapid and reproducible exacerbation of pruritus within seconds of acute emotional stress, consistent with rapid psychophysiologic modulation of symptom intensity. The reproducible association between stress and symptom exacerbation, together with the subsequent response to neuro-modulatory therapy after failure of antihistamines, H2 blockade, montelukast, and corticosteroids, provided strong clinical support for a centrally mediated neuro-immune mechanism underlying her condition. Overall, the disease course evolved over approximately four months from an acute inflammatory eruption to a chronic, centrally amplified pruritic disorder.

## Discussion

This case represents a clinically difficult and mechanistically important evolution from an acute arthropod-triggered inflammatory dermatosis to a chronic pruritic disorder driven by neuro-immune dysregulation. Although the index event was clinically consistent with bedbug exposure, with an initial eruption suggestive of arthropod bite hypersensitivity, the subsequent course did not behave like a purely histamine-mediated urticarial process. Despite a prolonged and adequate therapeutic trial of antihistamines, there was no meaningful clinical response, while corticosteroids provided only transient and partial improvement with rapid recurrence upon tapering. Most notably, the patient demonstrated a rapid and reproducible exacerbation of pruritus within seconds of acute emotional stress, indicating a strong and immediate psychophysiologic modulation of symptom intensity. In conjunction with evolving lesion morphology, persistent scratching, and short-lived therapeutic responses, these features collectively support a shift away from a predominantly histamine-mediated process toward a centrally amplified pruritic disorder.

The overall trajectory is best understood through the concept of a neuro-immune transition, in which an initial peripheral inflammatory trigger induces sustained neural sensitization and altered central itch processing. Formal quantitative sensory or small-fiber assessments were not performed. However, such investigations are not routinely part of standard clinical evaluation in chronic pruritus, and the mechanistic interpretation in this case is supported by a consistent clinical trajectory and therapeutic response [1-3].

In the early phase, the patient’s presentation was likely driven by classical immune activation involving mast cells, histamine release, and a Th2-skewed inflammatory response to arthropod antigens. However, the persistence and evolution of symptoms beyond the expected acute phase suggest a shift toward non-histaminergic itch pathways, which are increasingly recognized as dominant drivers of chronic pruritus. Cytokines such as interleukin-31 and epithelial-derived alarmins, including thymic stromal lymphopoietin, along with IL-4 and IL-13, are known to directly activate pruriceptive C-fibers through receptors expressed on primary sensory neurons, including IL-31 receptor A and the TSLP receptor complex, with downstream signaling mediated in part via JAK-STAT pathways. These mediators also sensitize transient receptor potential ion channels, particularly TRPV1 and TRPA1, lowering activation thresholds and amplifying neuronal excitability. In this context, itch is no longer dependent on histamine alone but is propagated through cytokine-driven, neuropeptide-mediated, and ion channel-dependent pathways that remain active despite aggressive H1 blockade [1-3].

This mechanistic framework explains the limited and unsustained efficacy of antihistamines in this patient. Histamine-dependent itch represents only one pathway of pruritus, and chronic itch frequently becomes dominated by histamine-independent circuits. It also explains why montelukast failed. Montelukast selectively inhibits cysteinyl-leukotriene receptor 1 and is most useful when leukotriene-driven eosinophilic or allergic inflammation is a major component of disease biology. In this patient, however, the condition evolved into a phenotype dominated by IL-31, TSLP, neuropeptide, and TRP-channel-mediated signalling rather than persistent leukotriene-mediated inflammation. Once such pathways predominate, leukotriene antagonism is expected to provide little clinical benefit. In that sense, montelukast did not fail because the disease was globally resistant to therapy, but because it was targeting the wrong biological pathway in the later phase of illness. This distinction is clinically important because many chronic itch patients continue to receive escalating anti-allergic therapy even after the disease has shifted away from allergy-dominant mechanisms [3,8].

Repeated peripheral stimulation in such cases leads to peripheral sensitization, characterized by increased expression of neuropeptides such as substance P and calcitonin gene-related peptide, together with enhanced production of nerve growth factor. Nerve growth factor promotes epidermal nerve fiber proliferation and hyperinnervation, increasing the density and sensitivity of cutaneous sensory endings. Sustained afferent input then drives central sensitization within the dorsal horn of the spinal cord, involving enhanced excitatory synaptic transmission, increased NMDA receptor activity, reduced inhibitory GABAergic tone, and recruitment of itch-specific spinal interneuronal circuits. These changes result in persistent neuronal hyperexcitability and altered sensory gain, clinically manifesting as exaggerated itch responses to minimal stimuli and amplification of the itch-scratch cycle. In this patient, scratching consistently provoked further itch in a reproducible manner, supporting an alloknesis-like phenomenon and reflecting disordered central processing rather than purely peripheral inflammation. In this state, pruritus begins to resemble chronic pain in its neurobiological behavior: although the initiating peripheral trigger remains relevant, symptom persistence is increasingly driven by maladaptive central processing and reinforcement loops within spinal and supraspinal networks [1-3,9].

The corticosteroid story in this case is particularly revealing. The patient repeatedly achieved only temporary benefit from systemic corticosteroids, including during a prolonged taper, followed by recurrence as doses were reduced. That pattern suggests suppression of the inflammatory surface component without durable reversal of the underlying neural sensitization. Corticosteroids can transiently dampen cytokine signalling and mast-cell activity, but they do not adequately reset dorsal horn sensitization, cortical salience processing, or the learned behavioural circuitry of the itch-scratch cycle. Recent integrative work on the itch-scratch cycle emphasizes that repetitive scratching perpetuates barrier injury, local inflammation, and central reinforcement, making relapse likely when treatment addresses only the inflammatory layer [9,10].

The question of escalation to biologic therapy becomes particularly relevant at this stage, given the apparent refractoriness to conventional treatment. Agents such as omalizumab and dupilumab are well-established in chronic spontaneous urticaria and type 2 inflammatory dermatoses, respectively, and would represent a logical next step in a persistently symptomatic patient [2,3]. However, the evolving clinical phenotype in this case suggested a shift away from a predominantly mast cell- or type 2 inflammation-driven process. The lack of sustained response to antihistamines, leukotriene receptor antagonism, and corticosteroids, together with the progressive dominance of excoriated lesions, persistent background pruritus, and strong psychophysiologic modulation, pointed toward a centrally amplified, neurogenic mechanism rather than ongoing peripheral immune activation. This distinction is particularly important in real-world practice, where refractory urticaria is often treated with escalation to immunologic therapy without reassessment of underlying disease biology.

In this context, escalation to biologic therapy was consciously deferred, as such agents primarily target upstream inflammatory pathways and may have limited efficacy once central sensitization and neuroimmune dysregulation become the dominant drivers of disease [1,9]. Instead, a neuro-modulatory approach was prioritized, resulting in clinically meaningful improvement. This highlights the importance of phenotype recognition and mechanism-based therapy in chronic pruritus and suggests that early identification of a neurogenic shift may prevent unnecessary exposure to high-cost biologic therapies with uncertain benefit.

A major strength of this case is that it also demonstrates how psychophysiologic amplification can become a principal determinant of disease severity. The patient repeatedly observed that the rash and itch worsened when she was “not okay,” and her clinical course confirmed that symptom intensity closely tracked emotional stress, occupational overload, frequent travel, interpersonal adversity, and likely sleep disruption. This is not merely a subjective overlay. Chronic stress alters both skin biology and itch perception through several pathways, including hypothalamic-pituitary-adrenal axis dysregulation, altered cortisol rhythms, sympathetic overactivation, enhanced mast cell-nerve cross-talk, and increased production of inflammatory mediators such as IL-6 and TNF-α. Stress also lowers central inhibitory control over somatic signalling and increases attentional capture by unpleasant bodily sensations, thereby increasing itch intensity even when peripheral lesion burden is modest. Recent work has shown measurable associations between perceived stress and chronic pruritus severity, reinforcing the biologic significance of stress in such patients [10,11].

The presence of ADHD in this patient adds an important neurobiological dimension and likely acted as an amplifying substrate rather than a coincidental comorbidity. Emerging literature links chronic pruritus, skin-picking behaviour, and ADHD-related symptom clusters, suggesting overlapping abnormalities in attentional control, inhibitory regulation, sensory salience, and stress responsiveness. Neurobiological studies, including functional neuroimaging, have demonstrated that ADHD involves dysregulation of dopaminergic and noradrenergic systems, particularly within prefrontal-striatal circuits that are critical for top-down inhibitory control and sensory gating [12]. This may predispose affected individuals to increased salience of somatic sensations, reduced ability to suppress urge-driven scratching, and greater behavioural reinforcement of the itch-scratch loop. The patient’s description of itch as intrusive, mentally consuming, and difficult to disengage from is highly consistent with this neurobehavioral model. A recent 2025 narrative review and case series specifically examining chronic pruritus, ADHD, and skin picking further supports the relevance of this association [6].

**Figure 1:**
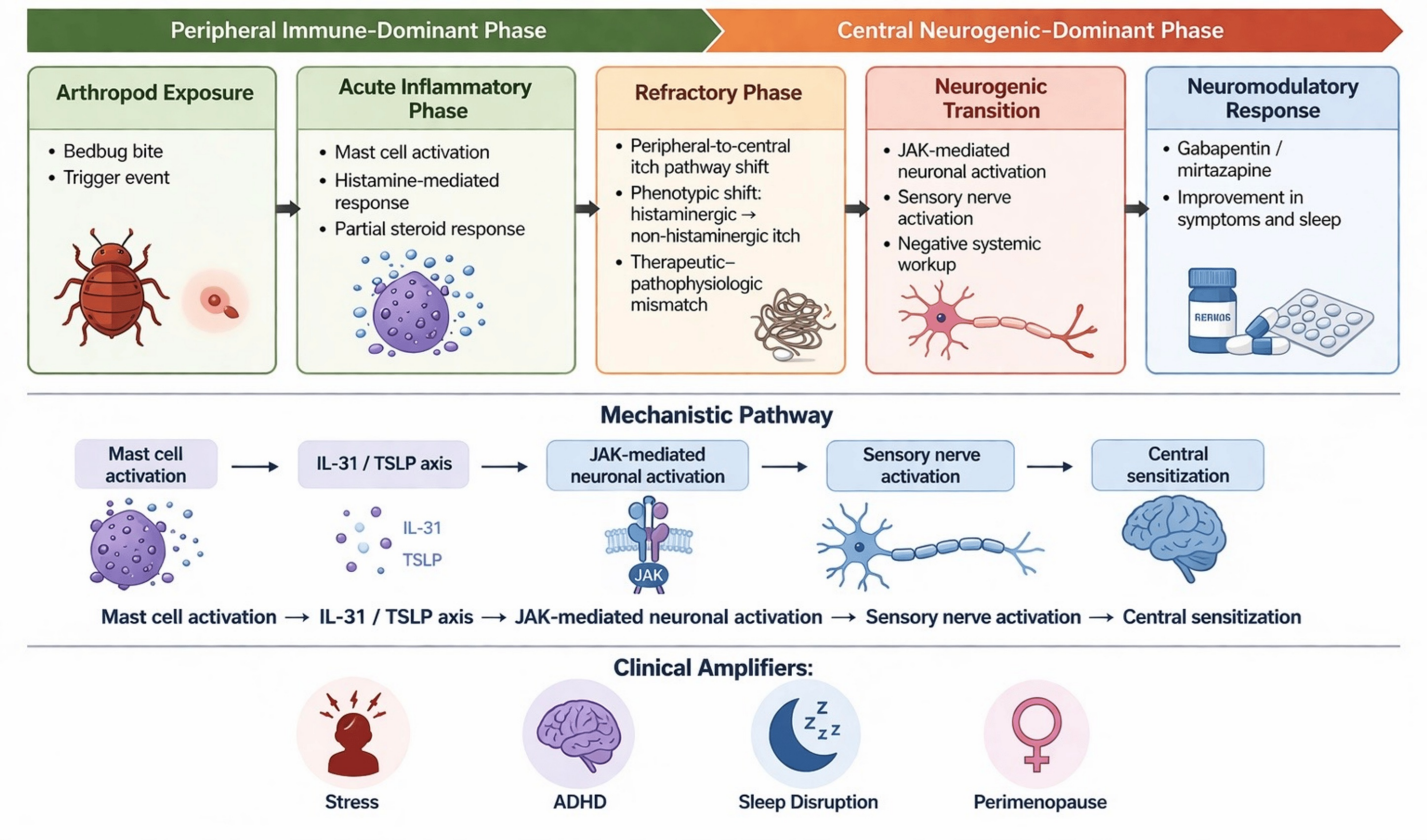
Neuroimmune transition in chronic pruritus Clinical trajectory illustrating the transition from peripheral immune-mediated inflammation following arthropod exposure to centrally amplified neurogenic pruritus, highlighting the evolving therapeutic response, neuroimmune pathways, and key clinical amplifiers. Created with Biorender.com

The patient’s menopausal state and alteration in hormonal milieu due to discontinuation of HRT may have contributed to increased cutaneous sensitivity and neuroimmune modulation, representing a plausible amplifying factor in disease evolution. Declining and fluctuating estrogen levels in midlife are associated with reduced epidermal lipid production, impaired barrier integrity, increased trans epidermal water loss, reduced glycosaminoglycans and collagen support, and heightened xerosis-associated pruritus. Estrogen also modulates mast-cell behaviour, serotonergic signalling, and GABAergic inhibitory tone, meaning that hormonal fluctuation can influence both peripheral cutaneous sensitivity and central sensory amplification. In practical terms, perimenopause may lower the threshold for itch generation while simultaneously making skin less resilient to repeated scratching. Recent systematic and clinical reviews support the idea that perimenopause is not merely a background context but a biologically relevant risk amplifier for chronic itch states [7,13]. The clinical trajectory and neuroimmune transition are summarized in Figure 1.

The early ecchymotic lesions in this case substantially influenced the diagnostic pathway and appropriately prompted evaluation for coagulation, thrombophilic, autoimmune, and vasculitic disorders. This broadened workup, including protein C, protein S, von Willebrand factor, factor assays, thrombophilia markers, and autoimmune serologies, was clinically justified at the time. In retrospect, however, the absence of meaningful laboratory abnormalities and the distribution of lesions strongly support a secondary mechanical etiology due to repetitive scratching in inflamed and sensitized skin, possibly compounded by local capillary fragility. This feature is academically useful because it shows how severe pruritic disorders can transiently mimic vasculitic or coagulation pathology and how a careful negative workup can meaningfully narrow the differential diagnosis rather than merely exclude disease.

The genetic and biologic predisposition component of this case also deserves attention, even though the patient was not genotyped. Current translational itch research suggests that individual susceptibility to chronic pruritus may be influenced by variation in IL31 and IL31RA signalling, TRPV1 and TRPA1 channel biology, neurotrophin pathways, such as nerve growth factor signalling, epidermal barrier genes, and genes involved in catecholamine regulation and stress responsivity. Such predispositions may help explain why a common trigger, such as arthropod bites, resolves uneventfully in most individuals but progresses to prolonged neurogenic pruritus in a smaller subset. This case does not prove a specific genetic driver, but it fits the emerging model that chronic pruritus reflects the interaction between a peripheral trigger and an underlying biologic susceptibility architecture [1,3].

The eventual improvement with gabapentin and mirtazapine is highly coherent with this framework and provides functional support for a centrally mediated mechanism. Gabapentin reduces neuronal excitability by binding the α2δ subunit of voltage-gated calcium channels, thereby decreasing the release of glutamate and other excitatory mediators involved in central sensitization. Mirtazapine adds benefit through central noradrenergic and serotonergic modulation, improvement in sleep, reduction in hyperarousal, and recognized antipruritic effects in chronic itch syndromes. Their effectiveness does not merely represent empirical symptom control; it serves as therapeutic confirmation that the disease had become centrally amplified and that distinguishing it from refractory urticaria mattered directly for management and outcome. Recent evidence syntheses support the therapeutic value of gabapentin in chronic pruritus, while mirtazapine continues to be recognized as a useful off-label option in centrally amplified itch states [14,15].

With regard to the broader literature context, global work increasingly supports neuroimmune models of chronic pruritus and recognizes the inadequacy of a purely antihistamine-centered approach in many chronic itch phenotypes. In contrast, literature from the Gulf and wider Middle East remains relatively sparse, particularly for mechanism-rich case reports linking arthropod-triggered eruptions, central sensitization, ADHD-related sensory amplification, and perimenopausal vulnerability. Broader reviews of pruritic inflammatory dermatoses in Arabic populations have also emphasized the need for more region-specific molecular, genetic, and biomarker data. That relative regional scarcity strengthens the educational value of this case, even if the underlying biology is globally relevant [16].

Taken together, this patient’s course is best conceptualized as a layered neuro-immune syndrome: an arthropod-triggered inflammatory eruption that evolved into chronic centrally amplified pruritus in a biologically susceptible host, with symptom severity magnified by ADHD-related sensory salience, chronic psychosocial stress, sleep disruption, and perimenopausal barrier and neurohormonal changes. Recognizing that transition was clinically decisive, because it shifted management away from repeated anti-allergic escalation and toward neuro-modulatory and psychophysiologic treatment, likely preventing unnecessary progression to biologic therapy for a condition that was no longer primarily mast-cell or histamine driven.

Learning points

This case highlights that chronic pruritus should not be assumed to remain histamine-mediated throughout its course, particularly when symptoms persist despite adequate antihistamine therapy and lesion morphology evolves away from transient wheals.

A rapid and reproducible relationship between emotional stress and symptom exacerbation, especially when occurring within seconds, may indicate centrally mediated modulation of itch and should prompt reconsideration of underlying mechanisms.

The presence of persistent scratching, excoriated papules, and post-inflammatory skin changes, along with short-lived or incomplete responses to corticosteroids, should raise suspicion for a transition toward a centrally amplified pruritic state.

Recognition of this evolving phenotype is clinically important, as it supports a shift from conventional anti-allergic therapies toward neuromodulatory and psychophysiologic approaches, potentially avoiding unnecessary escalation to biologic treatments.

Early recognition of such neuro-immune transitions may significantly shorten the disease course and reduce exposure to ineffective therapies.

## Conclusions

This case highlights a clinically significant evolution of chronic pruritus from an acute inflammatory trigger to a phenotype consistent with centrally amplified and neuro-immune-mediated itch. Recognition of this transition is critical, as it necessitates a shift from conventional anti-allergic strategies toward neuromodulatory and psychophysiologic approaches. In this patient, the combination of persistent non-response to histamine-targeted therapies, evolving lesion morphology, and a striking, rapid, and reproducible stress-associated exacerbation of symptoms provided strong clinical support for centrally driven modulation of itch perception. The sustained and clinically meaningful response to neuromodulatory therapy further supports this interpretation.

While the underlying mechanisms cannot be directly demonstrated in the absence of objective neurophysiological testing, the overall clinical trajectory is highly consistent with a neuro-immune transition involving peripheral sensitization and central amplification. This case underscores the importance of recognizing such evolving itch phenotypes in clinical practice to avoid unnecessary therapeutic escalation and to guide more targeted management strategies.
